# Environmental enrichment as a therapeutic strategy against methamphetamine induces depressive behaviors in mice

**DOI:** 10.1371/journal.pone.0333626

**Published:** 2025-10-03

**Authors:** Hammad Ismail, Syed Abdul Rafay, Talha Yaseen, Dania Khalid, Muhammad Zeeshan Bhatti, Salman Shahzad, Sabir Shahzad, Samreen Saleem, Abdur Rahman, Modhi O. Alotaibi, Areej Turkistani, Bushra Akram, Gaber El-Saber Batiha

**Affiliations:** 1 Department of Biochemistry and Biotechnology, University of Gujrat, Gujrat, Pakistan; 2 Department of Biological Sciences, National University of Medical Sciences, Rawalpindi, Pakistan; 3 Institute of Clinical Psychology, University of Karachi, Karachi, Pakistan; 4 Department of Human Nutrition & Dietetics, Faculty of Allied Health Sciences, Health Services Academy, Islamabad, Pakistan; 5 Punjab University College of Pharmacy, University of the Punjab, Lahore, Pakistan; 6 Department of Biology, College of Science, Princess Nourah bint Abdulrahman University, Riyadh, Saudi Arabia; 7 Environmental and biomaterial Unit, Natural and Health Sciences Research Center, Princess Nourah bint Abdulrahman University, Riyadh, Saudi Arabia; 8 Department of Pharmacology and Toxicology, Collage of Medicine, Taif University, Taif, Kingdom of Saudi Arabia; 9 Department of Psychology, University of Gujrat, Gujrat, Pakistan; 10 Department of Pharmacology and Therapeutics, Faculty of Veterinary Medicine, Damanhour University, Damanhour, AlBeheira, Egypt; Nathan S Kline Institute, UNITED STATES OF AMERICA

## Abstract

Methamphetamine (METH) abuse is associated with addiction, emotional dysregulation, motor impairments, cognitive deficits, and depressive symptoms, which are exacerbated by stress and social isolation. This study investigated the therapeutic potential of combined treatment and environmental enrichment in METH withdrawn mice. Behavioral, biochemical and neurochemical outcomes were evaluated. Post-treatment results demonstrated: motor and exploratory behavior *via* OFT (open field test) mobility increased to from 32.37 meters (withdrawal group) to 59.52 meters, anxiety reduction indicated in EPM (elevated plus maze test) open arm rose from 33.2 to 74.2 sec, while cognitive improvement by MWM (morris water maze test) escape latency decreased from 225.4 to 127.4 sec in hidden platform trial. Moreover, memory and exploration activity were determined by NOR (novel object recognition test) and HBT (hole board test) which showed enhanced novel object exploration (16.4 vs 5 sec) and exploratory behavior (17.6 explored areas vs 8.6). Social interaction time increased to 41.64 sec, and light-dark compartment preference improved to 68.4%. Biochemical analysis revealed elevated antioxidants enzymes activity POD (peroxidase) (0.178 U/min) SOD (superoxide dismutase) (0.82 U/mg protein) and CAT (catalase) (3.6 U/min) indicating improved oxidative stress resilience. Neurotransmitters restoration was observed with serotonin (0.21 µg/mg) and dopamine (0.46 µg/mg) levels rebounding from negligible withdrawn group. These findings demonstrate that combined treatment and enriched environment robustly accelerate functional recovery in METH-withdrawn mice, suggesting a promising strategy for mitigating addiction-related deficits.

## Introduction

Methamphetamine (commonly referred to as “METH”) is a synthetic psychostimulant with profound effects on the central nervous system. Known chemically as N-methylamphetamine, it belongs to the amphetamine family of compounds and is characterized by its high abuse potential and devastating health consequences [1]. First synthesized in 1893 while more potent crystalline form, known as crystal METH, was developed in 1919. Initially used for therapeutic purposes, its stimulant properties were exploited during World War II to enhance soldier alertness and endurance [2]. Methamphetamine’s use spans both licit and illicit contexts. Medically, it has been prescribed in limited cases, such as the treatment of attention deficit hyperactivity disorder (ADHD) and obesity [3]. However, its illicit production and recreational use have transformed it into a global public health crisis.

Methamphetamine acts primarily by increasing the release and blocking the reuptake of dopamine, serotonin, and norepinephrine in the brain. This results in heightened stimulation of the reward pathways, producing euphoria, increased energy, and hyperfocus [4]. Unlike amphetamine, methamphetamine is highly lipid-soluble, allowing it to cross the blood-brain barrier rapidly and exert more pronounced effects [5]. This rapid onset of action contributes significantly to its addictive potential. It is commonly used *via* smoking, snorting, injecting, or ingesting, with each route of administration influencing the drug’s onset and intensity of effects [6]. Recreational users often engage in binge patterns, consuming the drug repeatedly over several days to maintain its effects. Chronic use leads to tolerance, necessitating higher doses to achieve the same effects, thereby increasing the risk of addiction and overdose [7].

Neurotoxicity is a hallmark of chronic methamphetamine use, characterized by significant damage to dopaminergic and serotonergic neurons, which can result in cognitive deficits, memory impairment, and mood disorders [8]. During METH withdrawal, studies have observed that anxiety and depression-like behaviors increase significantly in animal models. The environment plays a crucial role in both the onset and exacerbation of METH-induced depression. Environmental stressors, in combination with METH’s impact on brain chemistry, can lead to a vicious cycle where emotional and psychological stress further worsens the depressive state [9].

Methamphetamine remains a significant global challenge, with its widespread abuse causing severe health, social, and economic consequences. Effective treatment modalities include behavioral therapies such as cognitive-behavioral therapy (CBT) and contingency management, which provide rewards for positive behaviors, while pharmacological interventions remain limited [10]. These therapies help individuals overcome negative thoughts and create a supportive environment conducive to recovery. However, a comprehensive approach involving prevention, treatment, and regulation is essential to mitigate the impact of methamphetamine abuse. This study aims to evaluate the effects of drug treatment and an enriched environment on methamphetamine (METH) addicts in mice following withdrawal. In this study, we hypothesize that environmental enrichment can alleviate methamphetamine-induced depressive-like behaviors in mice during withdrawal. We further postulate that the combination of environmental enrichment and therapeutic interventions will have a synergistic effect, leading to a greater reduction in depressive behaviors compared to standard conditions.

## Materials and methods

### Animal models

Albino mice (female) were taken for the study which were fed on their regular diet ad libitum. The study design was accepted by the Institutional Animal Ethics Committee of University of Gujrat (IRB No 320), and all provisions were carried to reduce animal sufferings. Anesthesia was administered using an intraperitoneal injection of ketamine (100 mg/kg) and xylazine (10 mg/kg) prior to any procedures that could cause discomfort. At the end of the experiment, animals were humanely euthanized using a high-dose injection of sodium pentobarbital (150 mg/kg, i.p.), followed by cervical dislocation to ensure death. All efforts were made to minimize pain and distress throughout the study. The mice were divided into six groups each containing five mice.

Animals were randomly assigned to experimental groups, and investigators conducting behavioral assessments and data analysis were blinded to treatment conditions. These groups were normal Saline (5 ml/kg saline), METH (5 mg/kg/day methamphetamine drug), WTD (withdrawal from drug and no treatment), WTD + TRT (withdrawal + Epival (5 mg/kg) + Risperidone (0.05 mg/kg) and WTD + TRT+ENVIR (withdrawal + Epival (5 mg/kg) + Risperidone (0.05 mg/kg) + Environment). The enriched environment was provided to mice by addition of toys, wooden swing, plastic tubes, running wheels, colorful balls, two story cage and climbing slide.

### Administration of methamphetamine

Methamphetamine was obtained from Sigma-Aldrich. Dilutions of METH were prepared in saline and administered by intraperitoneal injection to mice in a concentration of 40 mg/kg in all treatment groups except saline. The treatment was administered after that for 21 days. Behavioral tests were performed after 14 days.

### Behavior tests

#### Open field test (OFT).

The open field test was conducted over a 30-minute period to assess the locomotor activity and anxiety-related behavior in mice [11]. Each mouse was placed in the center of a 50 × 50 cm open field arena with 40 cm high walls. The arena was divided into equally sized squares. The movements of the mice were recorded using an overhead camera system. Mobility rate, measured as the total distance traveled, and exploratory behavior, measured by the number of areas explored, were tracked. Immobility time, the duration of time the mouse remained motionless, was also recorded.

#### Elevated plus maze test (EPM).

The EPM test, conducted over 10 minutes, was used to assess anxiety-related behavior in mice [12]. The apparatus consisted of two open arms and two closed arms arranged in a plus shape. Each mouse was placed in the center of the maze, and its movements were tracked using an overhead camera. The primary measures included the time spent in open versus closed arms and the number of entries into each arm type. Data was analyzed to determine anxiety levels, with increased time in closed arms indicating higher anxiety.

#### Morris water maze test (MWM).

The MWM hidden test, lasting 10 minutes, was designed to evaluate the spatial memory and learning in mice [13]. Each mouse was placed in a water maze containing a platform. Mice underwent four trials per session, with the latency to locate the platform recorded for visible and hidden trails. During the probe trial, the platform was removed, and the time spent in the target quadrant, where the platform was previously located, was measured. Data were collected to assess the learning and memory retention capabilities of the mice.

#### Forced swim test (FST).

Forced swim test was conducted to evaluate mice mobility for its survival when mice were forced to swim in water [14]. The mice were let into 25 cm height glass cylinder where water was filled up to 10 cm. Each mice was put in cylinder individually for 10 minutes. The immobility time was noted as time when mice was just floating over water surface without any struggle to swim. Frozen events are time when mice doesn’t struggle to keep its mouth above water.

#### Hole board test (HBT).

The HBT was used to evaluate anxiety of mice using their natural ability of dipping the head [15]. A grey box of 40 x 40 cm was taken with width of 2 cm consisting of 16 holes each of 3 cm diameter. The mice were put on to the board for 10 minutes. The nose poking to explore new places and head dips were counted. The mobility time to move to holes and frozen events where no movement is observed were also noted.

#### Novel object recognition (NOR) test.

The NOR test, conducted over a 10-minute period, evaluated recognition memory in mice [16]. Initially, mice were habituated to an arena containing two identical objects. After a retention interval, one of the objects was replaced with a novel object. The time spent exploring each object during the test session was recorded. The discrimination index, calculated as the ratio of time spent on the novel object to the total exploration time, was used as a measure of recognition memory.

### Social interaction test

The social interaction between mice was evaluated to check their environment adaptability [17]. The mice were separated 24 hours before test. The number of mice’s contact with other mates and frozen events were measured when mice is not interacting with other mice. The time of contact and distance covered by mice over a period of 10 minutes was measured.

#### Light-dark compartment test.

The light dark compartment test was used to evaluate anxiolytic effects of drug on mice [18]. The white rectangular open box was attached to a dark closed chamber with a small opening having a dark lid for movement into light chamber. The mice were placed in the dark chamber and its movement towards light chamber was evaluated over 10 minutes. The mice were considered in light chamber when all four limbs were in it. Time spent in light chamber, transitions between two chambers and head pokes to light chamber were counted.

### Dissection of mice and tissue homogenization

The mice were dissected on 21^st^ day. The skull was opened and the brain cortex was excised and stored in formalin. The brain tissues were homogenized in 0.1 M phosphate buffer which was centrifuged at 1000 rpm for 15 minutes. The supernatant was collected and stored at −20°C for antioxidant analysis and HPLC.

### Antioxidant analysis

The brain tissues were subjected to analyze the antioxidant enzymes level on microplate reader at specified wavelengths for each enzyme. Catalase (CAT) levels were analyzed at 240 nm by Aiebe’s method and expressed as U/min [19]. Superoxide dismutase (SOD) levels were expressed as U/mg protein and evaluated on a wavelength of 560 nm [20]. Peroxidase (POD) levels were analyzed on 470 nm and expressed in U/min [21].

### HPLC for neurotransmitters detection

The neurotransmitters dopamine and serotonin were detected in brain homogenized tissue by HPLC. Samples were filtered using 0.2 µm Teflon syringe filter and then 20 µl of sample was injected into apparatus. The carbon-18 column was used with polar solvent mobile phase consisting of 50 mM phosphate buffer and methanol in 97:3 (v/v) ratio. The standards were run followed by sample. The flow rate was 1.5 ml/min with a run time of 15 minutes at 240 nm.

### Statistical analysis

Statistical analyses were performed using GraphPad Prism 8.0. Data are presented as mean ± SD. Differences among groups were assessed by one-way analysis of variance (ANOVA) followed by Tukey’s multiple comparison post hoc test. A p-value of < 0.05 was considered statistically significant. In the figures, groups not sharing the same letter are significantly different, whereas groups with at least one common letter are not significantly different. The letters correspond to the following groups: (a) SALINE, (b) METH, (c) WTD, (d) WTD + TRT, and (e) WTD + TRT+ENVIR.

## Results

### Open field test

**Fig 1:**
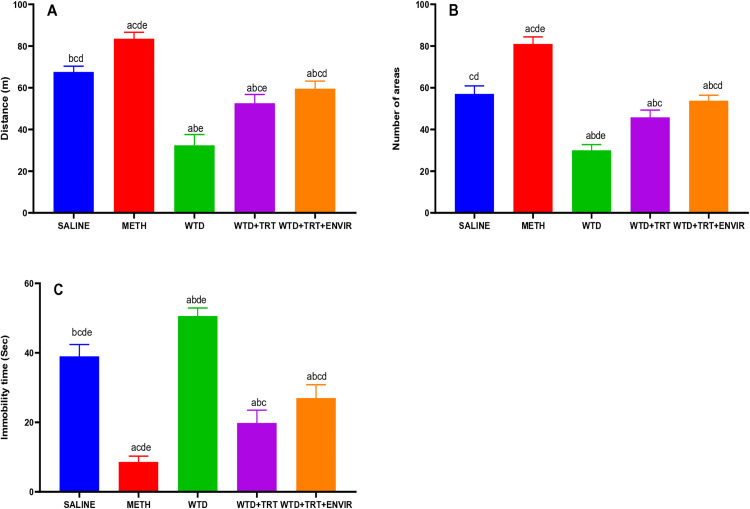
(A) Mobility distance (B) Explored areas, (C) Immobility time for open field test. Data are expressed as mean ± SD. Statistical analysis was performed using one-way ANOVA followed by Tukey’s multiple comparison test. Groups not sharing a common letter differ significantly (p < 0.05). The letters correspond to the following groups: (a) SALINE, (b) METH, (c) WTD, (d) WTD + TRT, and (e) WTD + TRT+ ENVIR.

The open field test was conducted to learn the effects of environmental enrichment on mice. The saline mice showed an average mobility rate of 67.59 ± 2.73 meters. In METH group, their mobility rate increased to 83.49 ± 3.12 meters, showing the significant effects of METH on the activity. WTD group showed mice became lazy as mobility rate dropped to 32.37 ± 5.23 meters. WTD + TRT increased their mobility rate to 52.56 ± 4.19 meters. Adding enriched environment further WTD + TRT+ ENVIR helped mice get better and mobility rate was recorded at 59.52 ± 3.67 meters as shown in Fig 1A. The saline mice showed an immobility time of 39 ± 3.39 sec. When METH was given to them, the immobility time recorded was 8.6 ± 1.67 sec, the least among all groups. In WTD group, withdrawing METH caused stress which ultimately led to an even increased immobility time 50.6 ± 2.3 sec. When treatment WTD + TRT was given, the immobility time recorded was less than saline 19.8 ± 3.7 sec. Environmental enrichment in addition to the treatment WTD + TRT+ENVIR increased the immobility time to 27 ± 3.8 sec as shown in Fig 1B. The number of areas explored by mice were calculated as shown in Fig 1C during the test. The saline group explored 57 ± 3.8 areas during the 30 minutes test period. After METH mice become more active, increasing explored areas to 81 ± 3.3 areas. When the METH was withdrawn in WTD group, the activity to explored area dropped to 30 ± 2.7 areas. WTD + TRT mice recovered from stress and explored areas increased to 45.8 ± 3.4 areas.

Open field test results indicated better locomotion and mobility activities after environmental enrichment compared to meth withdrawal group. In OFT, the results showed a significant decrease in mobility for the WTD group compared to the control group, which is consistent with previous studies demonstrating that poor nutrition and lack of intervention lead to a decline in locomotor activity. A study observed that nutrient-deprived rodents had a 40–50% reduction in mobility compared to controls [22], similar to 52% reduction seen in WTD group. About 18% increase in locomotion under methamphetamine treatment [23], aligning with METH group’s 24% increase compared to controls. WTD + TRT and WTD + TRT+ENVIR showed gradual improvement 53 and 60 meters respectively, corroborating study, where 20% recovery in mobility with environmental enrichment following neurodegenerative interventions [24]. Decreased exploratory behavior in nutrient-deficient models of 40% reduction is documented [25] compared to 47% decrease in the WTD group.

### Elevated plus maze test

**Fig 2:**
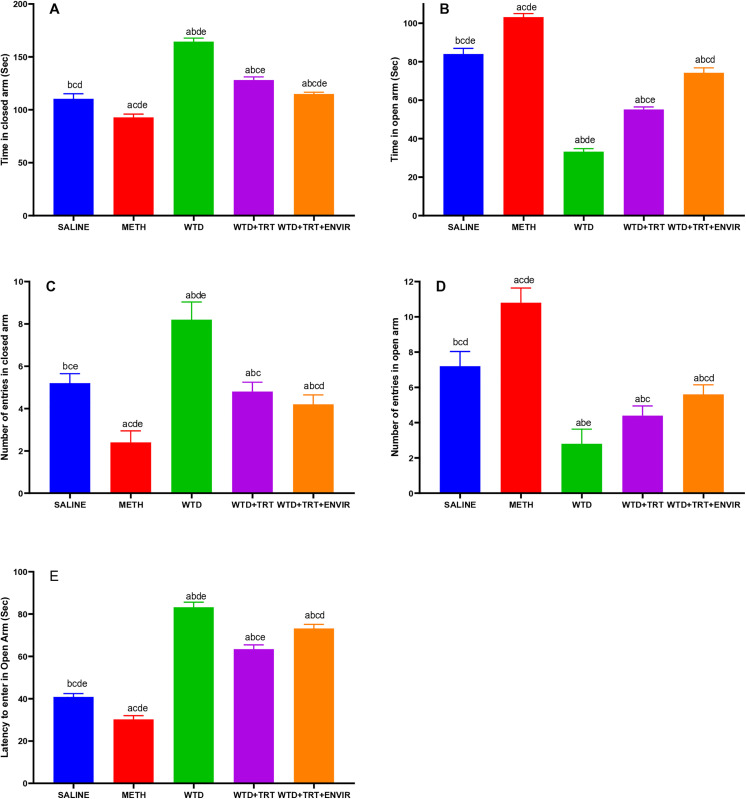
(A) Time spent in closed arm, (B) Time spent in open arm, (C) Number of entries in closed arm, (D) Number of entries in open arm, and (E) Latency time to enter open arm for elevated plus maze test. Data are expressed as mean ± SD. Statistical analysis was performed using one-way ANOVA followed by Tukey’s multiple comparison test. Groups not sharing a common letter differ significantly (p < 0.05). The letters correspond to the following groups: (a) SALINE, (b) METH, (c) WTD, (d) WTD + TRT, and (e) WTD + TRT+ ENVIR.

In elevated plus maze test, the anxiety level was observed as the anxiety increases when mice spend more time in closed arm compared to open arm as shown in Fig 2A and 2B. The control group saline exhibited a mean time in the closed arm of 110.4 ± 4.77 sec compared to 84 ± 2.91 sec for open arm. WTD group showed a mean time of 164.4 ± 3.36 sec and 33.2 ± 1.64 sec for closed and open arm. METH group exhibited a lower mean time in the closed arm at around 92.8 ± 3.11 sec, compared to 103.2 ± 1.78 sec for open arm suggesting a significant decrease in the time spent in the closed arm. WTD + TRT group recorded a mean time in the closed arm of 128.2 ± 2.86 sec and 55.2 ± 1.3 sec for open arm demonstrating an improvement over the WTD group in open arm. WTD + TRT+ENVIR group showed a mean time in the closed arm 115 ± 1.58 sec and 74.2 ± 2.58 sec in open arm.

The saline group exhibited a mean 5.2 ± 0.44 number of entries in the closed arm than to 7.2 ± 0.83 entries in open arm. WTD group showed a significantly higher entries 8.2 ± 0.83 compared to 2.8 ± 0.83 entries in open arm METH group exhibited the lowest mean number of entries of 2.4 ± 0.54, suggesting a significant decrease in the number of entries than to 10.8 ± 0.83 in open arm indicating less anxiety. The WTD with treatment (WTD + TRT) group recorded a mean number of entries of 4.8 ± 0.44 than 4.4 ± 0.54 entries in open arm. WTD + TRT+ENVIR group showed a mean number of entries of 4.2 ± 0.44 compared to 5.6 ± 0.54 entries for open arm indicating low anxiety as shown in Fig 2C and 2D.

Latency time is the time required to enter the open arm for first time indicating anxiety levels as shown in Fig 2E. The saline group exhibited a latency of 40.8 ± 1.64 sec. WTD group showed a significantly higher latency at 83.2 ± 2.38 sec, indicating a substantial increase in the time taken for the first visit to the open arm compared to the control group. METH group exhibited the lowest latency at 30.2 ± 1.78 sec, suggesting active state. WTD + TRT group recorded a latency of 63.4 ± 2.07 sec, WTD + TRT+ ENVIR group showed a latency of 73.2 ± 1.92 sec, which is higher than the control group’s level showing decreased anxiety levels.

EPM results showed increased time of mice in open arms after enriched environment exposure indicating diminished anxiety patterns. This study showed METH group’s immobility is reduced as latency time is less which aligns with previously recorded 75% decrease in immobility under methamphetamine exposure [26]. The more time spent in open arms and less latency time than withdrawal group is observed in the WTD + TRT+ ENVIR group which is comparable to López et al. 2022 where 50% recovery in anxiety metrics through environmental interventions was seen [27]. WTD group findings of increase in closed arm time are consistent documented 35–45% increases in anxiety-like behaviors in nutrient-deficient models [28].

### Morris Water Maze test

**Fig 3:**
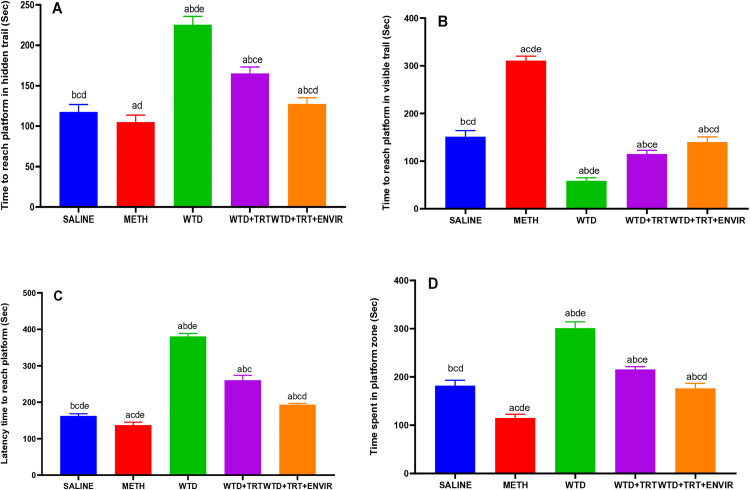
(A) Time taken to reach platform in hidden trail, (B) Time taken to reach platform in visible trail, (C) Latency time to reach platform zone and (D) Time spent in platform zone in probe trail for Morris water maze (MWM) test. Data are expressed as mean ± SD. Statistical analysis was performed using one-way ANOVA followed by Tukey’s multiple comparison test. Groups not sharing a common letter differ significantly (p < 0.05). The letters correspond to the following groups: (a) SALINE, (b) METH, (c) WTD, (d) WTD + TRT, and (e) WTD + TRT+ENVIR.

Morris water maze test was used to evaluate memory patterns. In the visible test, control group (saline) exhibited an average time 151.2 ± 12.7 sec. WTD group showed a significantly lower average time taken of 58.8 ± 6.26 sec, indicating a substantial decrease in the time taken compared to the control group. METH group exhibited the highest average time taken of 310.8 ± 9.39 sec, WTD + TRT group recorded an average time taken of 114.8 ± 7.98 sec, demonstrating an improvement over the WTD group. WTD + TRT+ ENVIR group showed further improvement with an average time taken of 140 ± 11.06 sec, although it remained below the control group’s level and difference was not statistically significant. In hidden test, the platform was not seen by the mice and mice reached based on their memory. saline group exhibited an average time taken of 117.6 ± 9.12 sec. WTD group showed a significantly higher average time taken of 225.4 ± 10.38 sec. METH group exhibited a lower average time taken at 105 ± 8.57 sec. WTD + TRT group recorded an average time taken of 165.2 ± 7.98 sec, demonstrating an improvement over the WTD group. WTD + TRT+ ENVIR group showed further improvement with an average time taken of 127.4 ± 7.66 sec. The results indicated improved memory as time for hidden trial was less than visible trial compared to Fig 3A and 3B.

Latency time was recorded and represented in Fig 3C. saline group exhibited an average latency time of 162.4 ± 5.85 sec. WTD group showed a significantly higher average latency time of 380.8 ± 7.98 sec. METH group exhibited the lowest average latency time at 137.2 ± 7.98 sec. WTD + TRT group recorded an average latency time of approximately 260.4 ± 13.46 sec, WTD + TRT+ ENVIR group showed further improvement with an average latency time of 193.2 ± 3.83 sec.

The control group (saline) exhibited an average platform zone time of 182 ± 11.06 sec. WTD group showed a significantly higher time of 301 ± 13.09 sec. METH group exhibited the lowest average platform time at 114.8 ± 7.98 sec, suggesting a significant decrease in the time taken. WTD + TRT group recorded time of 215.6 ± 5.85 sec. WTD + TRT+ ENVIR group showed further improvement with an average time spent in platform zone of 176.4 ± 10.38 sec, although it remained slightly above the control group’s level; this difference was not statistically significant shown in Fig 3D.

The spatial memory improved after exposure to better environment and platform location was retained in memory of mice in hidden and probe trials as they reached the platform point quickly after entering water. In this study, impaired spatial memory in WTD group showed increase in platform location time as 301 sec compared to 182 seconds of control which aligns with previous studies where 120–130% increase was observed in dietary deficiency models [29]. The time in platform zone decreases after METH exposure which was consistent with previous findings [30].

### Forced swim test

**Fig 4:**
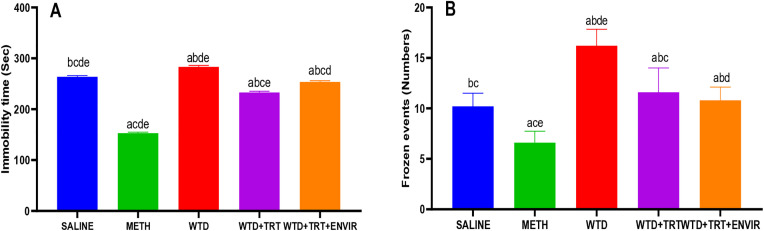
(A) Immobility time, (B) Frozen events for forced swim test. Data are expressed as mean ± SD. Statistical analysis was performed using one-way ANOVA followed by Tukey’s multiple comparison test. Groups not sharing a common letter differ significantly (p < 0.05). The letters correspond to the following groups: (a) SALINE, (b) METH, (c) WTD, (d) WTD + TRT, and (e) WTD + TRT+ ENVIR.

The forced swim test was conducted to study the behavior of mice by placing them in a water container, forcing them to swim. The saline group showed 263.6 ± 2.3 sec of immobility time. In METH group, mice became more active and immobility time was low 152.8 ± 1.92 sec. WTD group showed stress, due to which the immobility time was greater than the saline group 283.2 ± 2.86 sec. WTD + TRT, immobility time was recorded to be even lower than the saline 232.8 ± 2.38 sec. WTD + TRT+ ENVIR group showed an increase in the immobility time to 253.6 ± 2.3 sec shown in Fig 4A. The saline mice showed 10.2 ± 1.3 frozen events. In METH group, frozen events recorded was the lowest of all groups 6.6 ± 1.14 frozen events. In WTD group, an increase in the number of frozen events 16.2 ± 1.64, indicating signs of stress shown in Fig 4B. Our study observed that METH administration, withdrawal, treatment, and environmental enrichment significantly impacted immobility time and frozen events in the forced swim test. Treatment reduced immobility time compared to the saline and withdrawal group indicating antidepressant-like effects. Environmental enrichment, when combined with treatment moderated these effects and mice struggled for their survival. A study found that drug fluoxetine reduced immobility time, especially in enriched environments which was similar to our study where treatment following METH withdrawal led to a significant reduction in immobility time [31]. Additionally, [32] reported that imipramine (5 mg/kg) and fluoxetine reduced immobility time during repeated FST sessions, with environmental enrichment further enhancing the antidepressant effects, similar to our findings with treatment and environmental enrichment. Imipramine (5 mg/kg) also reduced swimming frequency, which was comparable to the reduction in frozen events observed in our study following METH withdrawal and treatment [32]. METH withdrawal shows increase of immobility time which is associated with depressive behavior. Immobility time was observed 220 sec in forced swimming test after METH administration was with-drawl which was similar to our study where WTD group showed 283 sec immobility time higher than saline [30].

### Hole board test

**Fig 5:**
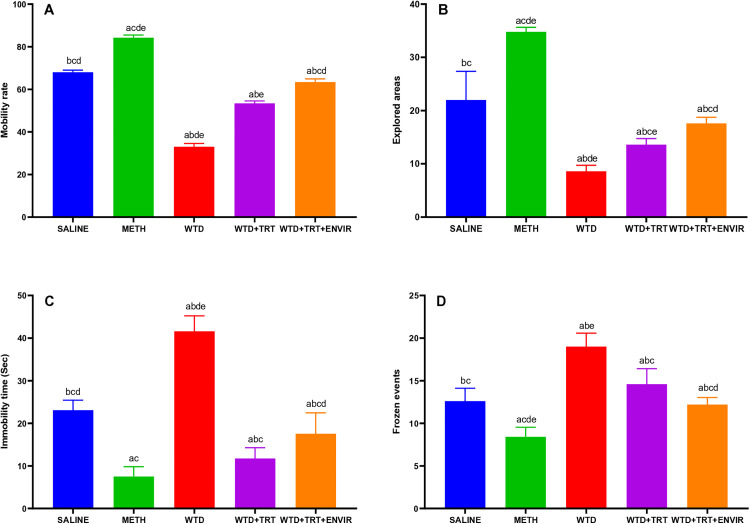
(A) Mobility rate, (B) Explored areas, (5C) Immobility time, (D) Frozen events for hole board test. Data are expressed as mean ± SD. Statistical analysis was performed using one-way ANOVA followed by Tukey’s multiple comparison test. Groups not sharing a common letter differ significantly (p < 0.05). The letters correspond to the following groups: (a) SALINE, (b) METH, (c) WTD, (d) WTD + TRT, and (e) WTD + TRT+ ENVIR.

In this test, interaction of the mice with holes was recorded to look for signs of stress in the animals. The normal saline group showed mobility rate of 68 ± 1 sec. METH group showed a significant increase in the mobility rate of 84.2 ± 1.3 sec. WTD group resulted in a decreased mobility rate, even lower than saline 33 ± 1.58 sec indicating stress. WTD + TRT group showed increased mobility rate to 53.4 ± 1.14 sec. Adding environmental enrichment WTD + TRT+ ENVIR further helped mice to recover from the stress and mobility rate got even better 63.4 ± 1.51 sec as shown in Fig 5A. The saline mice explored 22 ± 5.38 areas. Group METH resulted in an increase in number of explored areas 34.8 ± 0.83. In WTD group, explored areas drop to low number of areas 8.6 ± 1.14. Treatment administration (WTD + TRT) increased in the number of explored areas to 13.6 ± 1.14. Addition of environmental enrichment (WTD + TRT+ ENVIR) increased number of explored areas to 17.6 ± 1.14 but still lower to normal saline group indicated in Fig 5B.

Saline group showed 23.08 ± 2.33 sec of immobility as shown in Fig 5C. The immobility time was very short when METH was given 7.5 ± 2.32 sec, which is due to the hypermobility caused by METH. However, longest immobility time was recorded in WTD group, i.e., 41.6 ± 3.63 sec, which could be an indicator of stress in these METH-addicted mice. WTD + TRT group fight stress and resulted in decrease in immobility time 11.74 ± 2.53 sec. The number of frozen events (Fig 5D) was recorded with saline group showed 12.6 ± 1.51 frozen events. Giving METH to mice increased their activity level and number of frozen events decreased 8.4 ± 1.14. In WTD group, activity level dropped, mainly due to stress caused by withdrawal of METH, which increased frozen events 19 ± 1.58.

HBT results indicated that curiosity of mice increased after treatment and enriched environment and mice explored new areas. METH administration increased exploratory behavior, while withdrawal drastically reduced it. The reduction in immobility time during METH exposure reflects hyperactivity, whereas withdrawal significantly increased immobility due to stress. Comparably, [33] documented variations in exploration, such as head-dipping frequency, while [34] observed changes in time spent in specific zones, reflecting altered exploratory tendencies across conditions. METH exposure decreased the frequency of frozen events, while withdrawal increased them, indicating stress-induced behavioral inhibition.

### Novel object recognition (NOR) test

**Fig 6:**
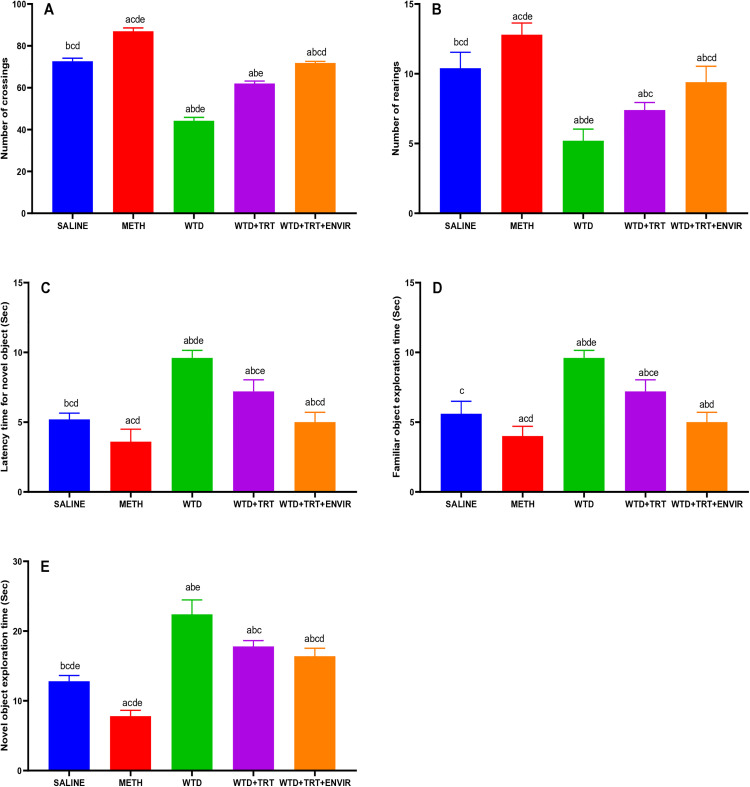
(A) Number of crossings, (B) Number of rearings, (C) Latency time for novel object, (D) Familiar object exploration time, (E) Novel object exploration time for novel object recognition test (NOR). Data are expressed as mean ± SD. Statistical analysis was performed using one-way ANOVA followed by Tukey’s multiple comparison test. Groups not sharing a common letter differ significantly (p < 0.05). The letters correspond to the following groups: (a) SALINE, (b) METH, (c) WTD, (d) WTD + TRT, and (e) WTD + TRT+ENVIR.

The mean number of crossings for mice across different experimental groups was measured to explore the curiosity among mice. Saline group exhibited a mean number of 72.6 ± 1.51 crossings, WTD group showed a significantly lower mean number of crossings (44.2 ± 1.64), indicating a substantial decrease in exploratory behavior compared to the control group. METH group exhibited the highest mean number of crossings (87 ± 1.58), suggesting a significant increase in exploration. WTD + TRT group recorded a mean 62 ± 1.22 number of crossings, demonstrating an improvement over the WTD group but still lower than the control group. The WTD + TRT+ENVIR group showed further improvement with a mean number 71.8 ± 0.83 of crossings however this difference was not statistically significant shown in Fig 6A.

The control group (saline) exhibited a mean number of rearings (10.4 ± 1.14). WTD group showed a significantly lower mean 5.2 ± 0.84 number of rearings, indicating a substantial decrease in exploratory behavior compared to the control group. METH group exhibited a higher mean 12.8 ± 0.83 number of rearings, suggesting a significant increase in exploration. WTD + TRT group recorded a mean 7.4 ± 0.54 number of rearings, demonstrating an improvement over the WTD group. WTD + TRT+ENVIR group showed further improvement with a mean 9.4 ± 1.14 number of rearings, which was comparable to the control group’s level shown in Fig 6B.

The control group (saline) exhibited a mean latency time of 5.2 ± 0.44 sec to explore any novel object in Fig 6C. WTD group showed a significantly higher mean latency time of 9.6 ± 0.55 sec, indicating a substantial increase in latency compared to the control group. METH group exhibited a lower mean latency time at 3.6 ± 0.89 sec, WTD + TRT group recorded a mean latency time of 7.2 ± 0.83 sec, demonstrating an improvement over the WTD group but still higher than the control group. WTD + TRT+ ENVIR group showed further improvement with a mean latency time 5 ± 0.71 sec.

The time to explore familiar object was calculated in Fig 6D. The control group (saline) exhibited a mean exploration time of 5.6 ± 0.89 sec. WTD group showed a significantly higher mean exploration time of 9.6 ± 0.55 sec, indicating a substantial increase in exploration time compared to the control group. METH group exhibited a lower mean exploration time at 4 ± 0.71 sec, WTD + TRT group recorded a mean exploration time of 7.2 ± 0.83 sec, demonstrating an improvement over the WTD group. WTD + TRT+ ENVIR group showed further improvement with a mean exploration time of 5 ± 0.71 sec.

The control group (saline) exhibited a mean exploration time of 12.8 ± 0.83 sec for a novel object. The WTD group showed a significantly higher mean exploration time of 22.4 ± 2.07 sec, indicating a substantial increase in exploration time compared to the control group. METH group exhibited the lowest mean exploration time at 7.8 ± 0.83 sec, suggesting a significant decrease in exploration time and increased curiosity. WTD + TRT group recorded a mean exploration time of 17.8 ± 0.83 sec, demonstrating an improvement over the WTD group. WTD + TRT+ ENVIR group showed similar results with a mean exploration time at 16.4 ± 1.14 sec, which was higher than the control group’s level indicating the existence of curiosity like behavior in mice after environment is better as indicated by Fig 6E.

NOR test results indicated that after treatment and exposure to better environments the mice were able to explore novel objects along with familiar objects which retained their curios and less anxious behavior which was low after meth withdrawal.

### Social interaction test

**Fig 7:**
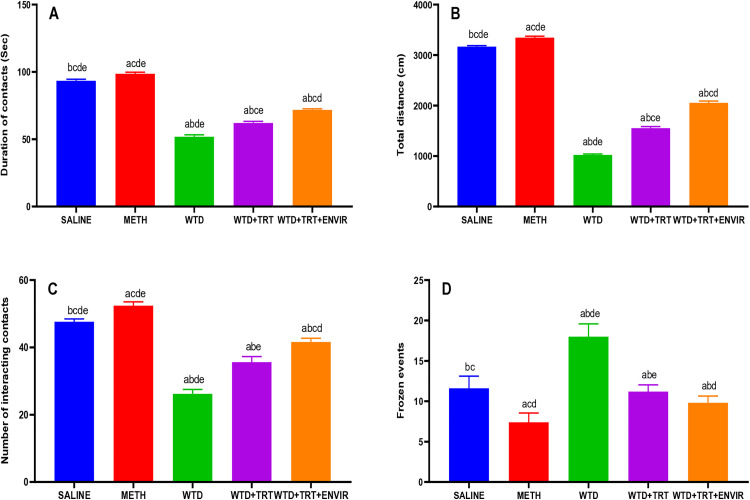
(A) Duration of contacts, (B) Distance covered, (C) Number of interacting contacts, (D) Number of frozen events for social interaction test. Data are expressed as mean ± SD. Statistical analysis was performed using one-way ANOVA followed by Tukey’s multiple comparison test. Groups not sharing a common letter differ significantly (p < 0.05). The letters correspond to the following groups: (a) SALINE, (b) METH, (c) WTD, (d) WTD + TRT, and (e) WTD + TRT+ ENVIR.

Social interaction test evaluates the interacting ability of mice after being isolated from their mates. The control group (saline) exhibited a total contact duration of 93.4 ± 1.14 sec. WTD group showed a significantly lower contact duration of 51.8 ± 1.48 sec, indicating a substantial decrease in social interaction. METH group exhibited the highest total contact duration at 98.6 ± 1.14 sec, suggesting a significant increase in social interaction. WTD + TRT group recorded a total contact duration of 62 ± 1.22 sec, demonstrating an improvement over the WTD group. WTD + TRT+ ENVIR group showed further improvement, with a total contact duration 71.8 ± 0.84 sec, which was closer to the control group’s level shown in Fig 7A.

The control group (saline) exhibited a total distance traveled of 3166 ± 20.7 cm. WTD group showed a significantly lower total distance of 1022 ± 19.2 cm, indicating a substantial reduction in locomotor activity compared to the control group. METH group exhibited the highest total distance traveled at 3346.4 ± 26.65 cm, suggesting a significant increase in locomotor activity. WTD + TRT group recorded a total distance of 1551.2 ± 30.34 cm, demonstrating an improvement over the WTD group but still lower than the control group. WTD + TRT+ ENVIR group showed further improvement with a total distance traveled around 2054 ± 33.62 cm shown in Fig 7B.

The control group (saline) exhibited a mean number of interacting contacts of 47.6 ± 0.89 between test mice with its mates shown in Fig 7C. WTD group showed a significantly lower number of contacts at 26.2 ± 1.30, indicating a substantial decrease in social interactions compared to the control group. METH group exhibited the highest number of contacts at 52.4 ± 1.14, suggesting a significant increase in social interactions. WTD + TRT group recorded a mean number of contacts of 35.6 ± 1.67, demonstrating an improvement over the WTD group but still lower than the control group. WTD + TRT+ ENVIR group showed further improvement with a mean number of interacting contacts of 41.6 ± 1.14.

Saline group exhibited a mean of 11.6 ± 1.51 frozen events. WTD group showed a significantly higher mean number of frozen events at 18 ± 1.58, indicating an increase in freezing behavior compared to the control group, which suggests heightened anxiety or fear. METH group exhibited the lowest mean number of frozen events at 7.4 ± 1.14, suggesting a significant reduction in freezing behavior, possibly due to reduced anxiety. WTD + TRT group recorded a mean number of frozen events of 11.2 ± 0.84, showing an improvement over the WTD group. WTD + TRT+ ENVIR group showed a further reduction with a mean of 9.8 ± 0.83 frozen events, which were closer to the control group’s level shown in Fig 7D.

The results from social interaction test indicate that high interacting and social ability was observed in mice exposed to enriched environment compared to those who were not exposed. In our study, environmental enrichment increased the contacts and distance travelled to 41 and 71 post treatment and environmental enriched conditions which was similar to previous studies where contact points of 30 and distance to 65 respectively were observed [35].

### Light-dark compartment test

**Fig 8:**
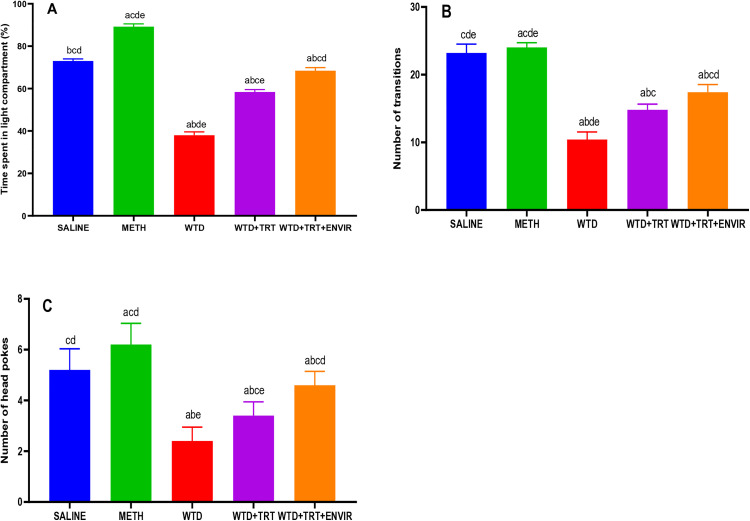
(A) Time spent in light compartment, (B) Number of transitions between compartments, (C) Number of head pokes to light compartment for light-dark compartment test. Data are expressed as mean ± SD. Statistical analysis was performed using one-way ANOVA followed by Tukey’s multiple comparison test. Groups not sharing a common letter differ significantly (p < 0.05). The letters correspond to the following groups: (a) SALINE, (b) METH, (c) WTD, (d) WTD + TRT, and (e) WTD + TRT+ENVIR.

In this test, the interaction of mice with light was used as a standard for evaluating the effects of stress on the behavior of mice. The saline group mice spent an average of 74 ± 1% of their time in the light compartment as indicated by Fig 8A. METH group showed an increase in their activity and curiosity, increasing the percentage of time spent in the light compartment 89.2 ± 1.3%. In WTD group, the mice went into a state of stress which was evident by a huge drop in the time spent in light compartment 38 ± 1.58% compared to saline. WTD + TRT group increased the time spent in light compartment as 58.4 ± 1.14%. Environment enrichment (WTD + TRT+ENVIR) increased the time spent in light compartment to 68.4 ± 1.51% which indicate better adaptability.

The number of transitions indicates how many times the mice moved from one compartment to the other as shown in Fig 8B. The saline mice were very active, showing 23.2 ± 1.3 transitions. In WTD group, the number of transitions dropped to almost half of that of the saline 10.4 ± 1.14 transitions. This could be related to the stress which caused them to spend more time in the dark compartment. Giving treatment (WTD + TRT) helped them to recover from the stress and the number. of transitions increased 14.8 ± 0.83. Environment enrichment (WTD + TRT+ ENVIR) to mice helped to lower the stress and increased the number of transitions to 17.4 ± 1.14.

Number of head pokes refers to the number of times the mice extent their head from the dark compartment into the light compartment without fully entering it, representing curiosity in the mice, results expressed in Fig 8C. In the test, the saline mice showed an average of 5.2 ± 0.83 head pokes. In WTD group, the no. of head pokes recorded were low 2.4 ± 0.54, indicating stress which decreased their curiosity. Giving treatment (WTD + TRT) increased the number of head pokes (3.4 ± 0.54).

The increased amount of time spent in light and movement towards light showed less stress and high adaptability in mice of enriched environment group. Significant behavioral changes were observed following various interventions, which align with those reported in earlier studies [36], demonstrated that diazepam treatment increased the time spent in the light compartment in BALB/c mice, reflecting reduced anxiety-like behavior. However, C57BL/6 mice did not show significant changes, suggesting strain-dependent responses to pharmacological treatment. These observations correspond with the trends noted in this study, where treatment and environmental enrichment effectively mitigated stress and anxiety-like behaviors [37], highlighted the effects of environmental enrichment, showing that animals housed under enriched conditions spent significantly more time in the light compartment, with increases of 1.2- to 1.4-fold compared to standard housing. This trend mirrors the increased exploratory behavior noted in this research under similar conditions. Transitions between compartments are indicative of locomotor and exploratory activity. Diazepam significantly increased the number of crossings in BALB/c mice, reducing anxiety-like behavior, whereas no significant effect was observed in C57BL/6 mice [36].

### Antioxidant analysis

**Fig 9:**
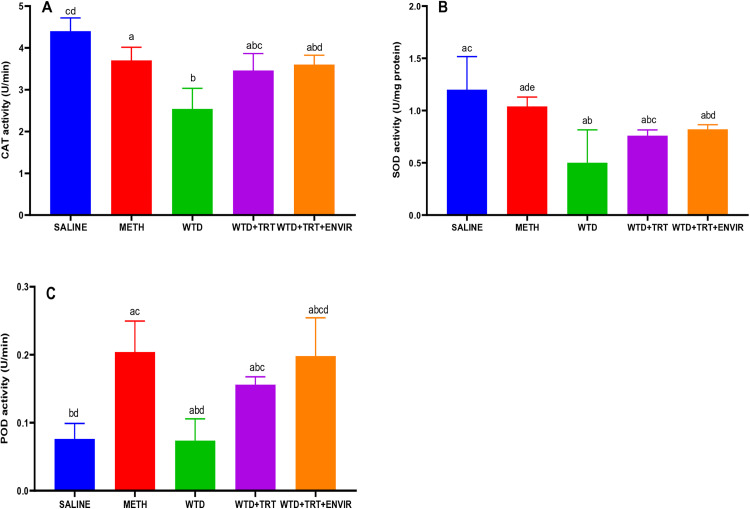
Levels of antioxidant enzymes (A) Catalase, (B) Superoxide dismutase, (C) peroxidase. Data are expressed as mean ± SD. Statistical analysis was performed using one-way ANOVA followed by Tukey’s multiple comparison test. Groups not sharing a common letter differ significantly (p < 0.05). The letters correspond to the following groups: (a) SALINE, (b) METH, (c) WTD, (d) WTD + TRT, and (e) WTD + TRT+ ENVIR.

The antioxidant test focuses on measuring the activity of the enzymes involved in the oxidative stress mechanism of the body. The activity of the enzymes can be correlated with the stress and can give information about the behavior of the mice. The activity of catalase (CAT), superoxide dismutase (SOD) and peroxidase (POD) enzymes was calculated in the brain samples as indicated in Fig 9.

The level of CAT (Fig 9A) in the saline mice was recorded at 4.4 ± 0.31 U/min. The level dropped with METH group 3.7 ± 0.31 U/min. This drop in the CAT activity indicate increase in the oxidative stress. In WTD group, lower activity 2.54 ± 0.49 U/min was seen, indicating an increase in the oxidative stress. When the mice were given treatment (WTD + TRT), it helped them to recover from the stress, which was shown by an increase in the CAT activity 3.46 ± 0.4 U/min. The CAT activity further increased when environmental enrichment (WTD + TRT+ENVIR) was added to the test 3.6 ± 0.22 U/min, showcasing the role of environmental enrichment in recovering from the stress.

The SOD activity (Fig 9B) was the highest in the saline mice with an average of 1.2 ± 0.31 U/mg protein. In WTD group, a huge drop in the SOD activity was noticed 0.5 ± 0.31 U/mg protein, which shows an increase in the oxidative stress in these METH-addicted mice. Treatment (WTD + TRT) was given which helped recover from the stress and the SOD activity increased 0.76 ± 0.05 U/mg protein. Addition of environmental enrichment (WTD + TRT+ ENVIR) further increased the SOD activity 0.82 ± 0.04 U/mg protein.

POD level (Fig 9C) was lowest in saline 0.076 ± 0.02 U/min. When METH group was given to mice, the activity of POD increased drastically 0.204 ± 0.04 U/min. After providing treatment (WTD + TRT), the activity of POD decreased 0.156 ± 0.01 U/min, showing a decrease in the oxidative stress. Addition of environmental enrichment (WTD + TRT+ ENVIR) caused an increase in the POD activity 0.178 ± 0.07 U/min, which could be related to the effect of environmental enrichment on the physiology of the animal.

The increase of enzymes in enriched environment group indicated the stress coping ability of these mice compared to meth withdrawal and no treatment group mice. The decline in catalase activity after METH exposure also mirrors patterns seen in other research, where oxidative stress caused by METH resulted in similar reductions in catalase levels, followed by recovery after intervention [38]. The environmental enrichment can reduce oxidative stress, with findings indicating a normalization of enzyme expression in treated animals. These findings are consistent with the partial recovery observed here, particularly in terms of SOD activity, after the addition of environmental enrichment [38]. In particular, the normalization of oxidative stress pathways following enrichment is evident in studies examining brain regions affected by chronic stress, such as the frontal cortex and brain stem, which parallels the trends observed in POD activity in this study [39].

### HPLC analysis

The level of neurotransmitters was calculated in the brain tissue samples of mice. The neurotransmitters were not detected in WTD group. In the saline group, the serotonin was recorded as 0.25 ± 0.002 µg/mg tissue. METH group showed the increased level of serotonin as 0.33 ± 0.005 µg/mg tissue. When treatment (WTD + TRT) was given, the observed level was lower than the normal value 0.19 ± 0.001 µg/mg tissue. Giving environmental enrichment (WTD + TRT+ ENVIR) to mice helped in improving serotonin levels 0.21 ± 0.005 µg/mg tissue.

**Fig 10:**
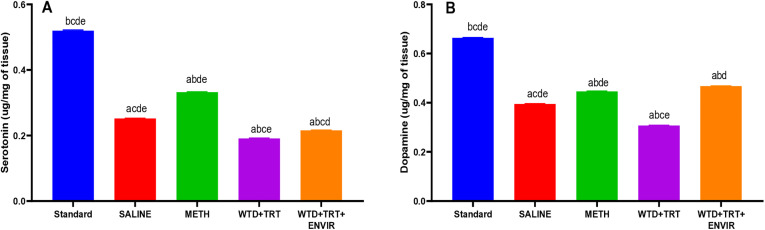
(A) Serotonin and (B) Dopamine levels in brain tissues. Data are expressed as mean ± SD. Statistical analysis was performed using one-way ANOVA followed by Tukey’s multiple comparison test. Groups not sharing a common letter differ significantly (p < 0.05). The letters correspond to the following groups: (a) SALINE, (b) METH, (c) WTD, (d) WTD + TRT, and (e) WTD + TRT+ENVIR.

The saline group showed an average value of 0.39 ± 0.001 µg/mg tissue for dopamine. However, administration of METH caused a significant increase in dopamine levels 0.44 ± 0.001 µg/mg tissue, which could be a reason for the increased activity of these mice. After treatment (WTD + TRT), dopamine levels decreased 0.31 ± 0.005 µg/mg tissue. Environmental enrichment with the treatment (WTD + TRT+ ENVIR) caused the dopamine levels to rise to 0.46 ± 0.0005 µg/mg tissue as shown in Fig 10.

The results indicate the magnificent decrease in levels after meth withdrawal which were restored close to normal control levels indicating better mood and motivation. The low level of neurotransmitters leads the individual to be in a depressive state as motivation and reward emotion decrease. The molecular underpinnings of depressive behaviors are tied to dysregulations in several key signaling pathways, most notably the Brain-Derived Neurotrophic Factor (BDNF)-ERK-CREB pathway. BDNF plays an essential role in the maintenance of neural circuits involved in mood regulation [40]. Chronic METH use leads to decreased levels of BDNF in critical regions like the hippocampus and prefrontal cortex, which correlates with the onset of depression-like behaviors during withdrawal [41]. Activation of BDNF triggers the ERK signaling cascade, which subsequently phosphorylates CREB, a transcription factor that regulates genes critical for synaptic plasticity and neuronal survival. Reduced ERK-CREB activity following METH exposure may impair synaptic remodeling and contribute to long-term mood disturbances. Environmental enrichment, however, has been reported to enhance BDNF expression and restore ERK-CREB signaling, potentially reversing METH-induced neuroadaptations. A study showed that environmental enrichment causes an increase in gene expression of neurotransmitters which was observed in this study as levels of dopamine and serotonin increased in WTD + TRT+ENVIR group [42]. The neurotransmitters dopamine and serotonin levels deplete after withdrawal of METH from mice which is similar to our results [43].

## Conclusions

The study exhibited that the treatment along with a better and enriched environment can be helpful for the METH-addicts to adapt to their normal life after withdrawal from its use. Results indicated that mice showed an improved performance and memory in behavioral tests for recognition in Morris water maze, open field test, forced swim test. The less anxious and more curios behavior was confirmed by elevated plus maze test, hole board test and novel objection recognition test. Adapting to surroundings and interaction with other mates was observed in social interaction and light-dark compartment test. Level of antioxidants and neurotransmitters was also elevated. The enriched environment proved to be an important factor for recovery of the METH addicted individuals. The results obtained from behavioral tests highlighted a statistically significant improved patterns of memory, alertness and curiosity in mice with enriched environment. The mice performed well and survived in new environments after subjection to enriched environmental conditions compared to control and no treatment groups. So enriched environment exposure proved to be a promising factor for better behavior of mice along with treatment administration.
